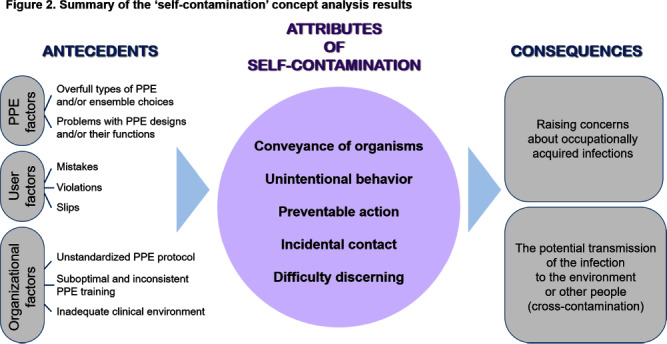# Self-contamination with use of personal protective equipment: Concept analysis using Rodgers’ evolutionary method

**DOI:** 10.1017/ash.2024.279

**Published:** 2024-09-16

**Authors:** JaHyun Kang, EunJo Kim

**Affiliations:** Seoul National University College of Nursing

## Abstract

**Background:** Although self-contamination has been identified through relevant studies on the safe utilization of personal protective equipment (PPE) by healthcare personnel and the prevention of healthcare-associated infection transmission, there is a lack of in-depth understanding of self-contamination. This study aimed to review previous research on self-contamination and clarify the concept. By understanding this concept, healthcare personnel can improve their conscientiousness regarding self-contamination and gain a foundation for the requisite interventions to reduce self-contamination in healthcare settings. **Method:** MeSH terms such as “Health Personnel,” “Students, Health Occupations,” and keywords related to PPE combined with “contaminat*” and “self-contamination” to retrieve literature in OVID, EMBASE, Cochrane, CINAHL, and PsycINFO databases without time limits through December 2023 (Figure 1). The concept of self-contamination’s antecedents, attributes, and consequences were explained based on Rodgers’ evolutionary **Results:** After eliminating duplicates from the 2,195 studies that were initially searched, the authors reviewed the articles and determined eligibility independently. A total of 25 articles, published between 2006 and 2023, were included and analyzed. Antecedents to self-contamination were classified into three groups for each component (Figure 2). First, PPE factors included 1) types of PPE and/or ensemble choices, and 2) problems with PPE designs and/or their functions. Second, user factors involved 1) mistakes (errors of intent), 2) violations (deviations from recommendations), and 3) slips (natural flaws in humans). Third, organizational factors covered 1) unstandardized PPE protocol, 2) suboptimal and inconsistent PPE training, and 3) inadequate clinical environment. In total, five major concept attributes were the conveyance of organisms, unintentional behavior, preventable action, incidental contact, and difficulty discerning. Consequences were categorized into: 1) raising concerns about occupationally acquired infections, and 2) the potential transmission of the infection to the environment or other people (cross-contamination). **Conclusion:** While studies have raised questions about whether self-contamination causes infection, self-contamination was obvious among healthcare personnel when dealing with PPE. Considering the wide range of causes and potential results of self-contamination, multifaceted interventions, including improvement of the PPE design, tailoring protocols, and training for specific ensembles, should be implemented over an extended time period with suitable intervals to optimize interventions’ effectiveness.

**Figure:**
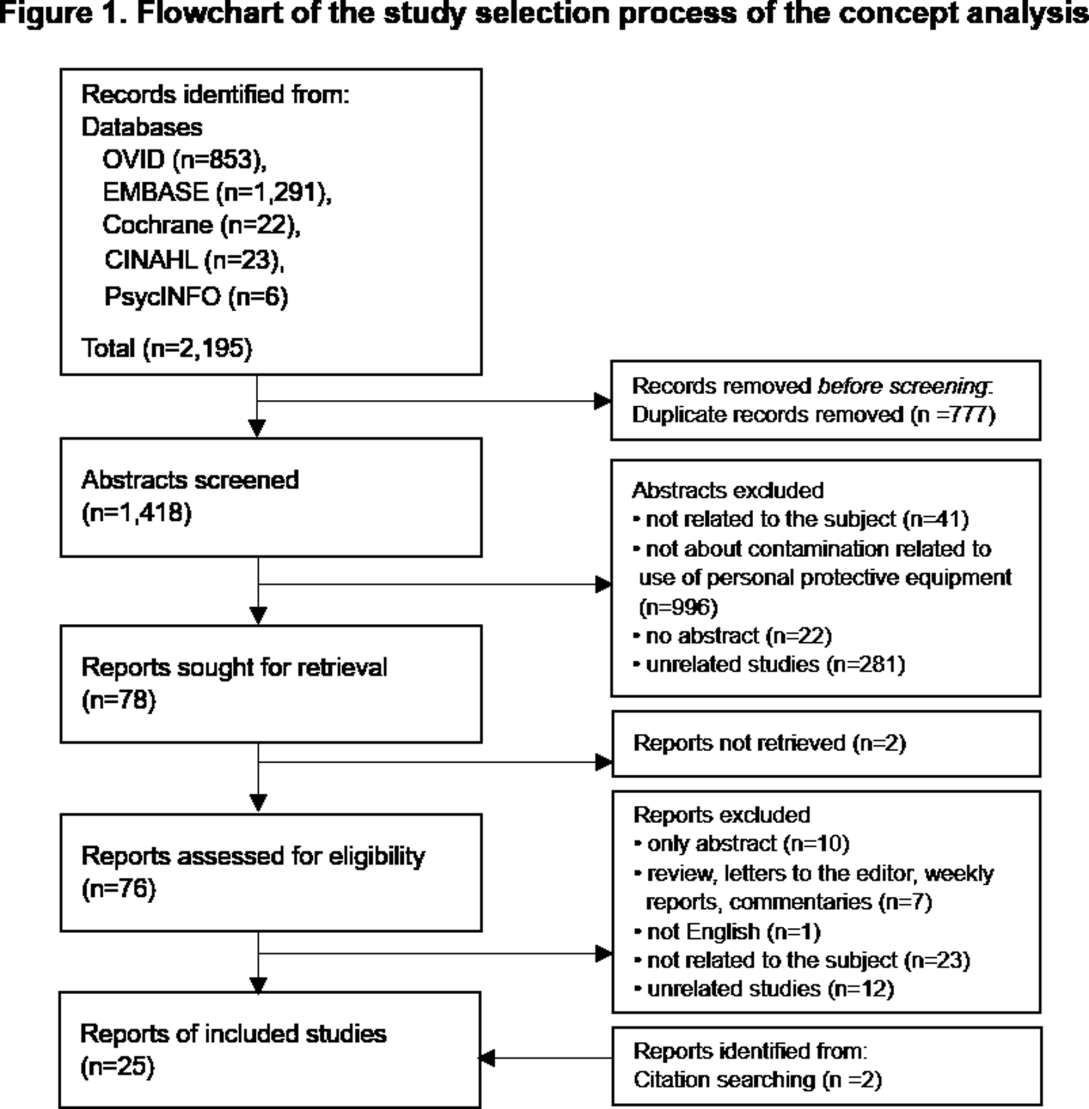

**Figure:**